# Self-organization of PIP3 waves is controlled by the topology and curvature of cell membranes

**DOI:** 10.1016/j.bpj.2024.03.022

**Published:** 2024-03-21

**Authors:** Sema Erisis, Marcel Hörning

**Affiliations:** 1 Institute of Biomaterials and Biomolecular Systems, University of Stuttgart, Stuttgart, Germany

## Abstract

Phosphatidylinositol (3,4,5)-trisphosphate (PIP3) is a signaling lipid on the plasma membrane that plays a fundamental role in cell signaling with a strong impact on cell physiology and diseases. It is responsible for the protruding edge formation, cell polarization, macropinocytosis, and other membrane remodeling dynamics in cells. It has been shown that the membrane confinement and curvature affects the wave formation of PIP3 and F-actin. But, even in the absence of F-actin, a complex self-organization of the spatiotemporal PIP3 waves is observed. In recent findings, we have shown that these waves can be guided and pinned on strongly bended *Dictyostelium* membranes caused by molecular crowding and curvature-limited diffusion. Based on these experimental findings, we investigate the spatiotemporal PIP3 wave dynamics on realistic three-dimensional cell-like membranes to explore the effect of curvature-limited diffusion, as observed experimentally. We use an established stochastic reaction-diffusion model with enzymatic Michaelis-Menten-type reactions that mimics the dynamics of *Dictyostelium* cells. As these cells mimic the three-dimensional shape and size observed experimentally, we found that the PIP3 wave directionality can be explained by a Hopf-like and a reverse periodic-doubling bifurcation for uniform diffusion and curvature-limited diffusion properties. Finally, we compare the results with recent experimental findings and discuss the discrepancy between the biological and numerical results.

## Significance

Lipid dynamics contribute to the adaptability of membranes, allowing cells to undergo shape changes during processes such as endocytosis and membrane fusion. They also affect the localization and activity of signaling proteins, and are therefore critical for maintaining cell integrity and responding to mechanical stress. Here, we introduce a realistic three-dimensional stochastic reaction-diffusion system that mimics experimentally observed PIP3 lipid dynamics on whole *Dictyostelium* cell membranes. The aim is to understand the fundamental biophysical dynamics at the whole-cell membrane and the role of the local membrane curvature-limited diffusion. The results suggest that curvature-limited diffusion is essential to explain the experimental results and, on a broader context, fundamental pattern formation in small confined excitable systems.

## Introduction

Many features of pathways observed in the model organism *Dictyostelium discoideum* are similar to those in eukaryotic cells (1), such as directional migration, cell adhesion, gene expression, and cell-cell signaling (2). In both organisms, the phosphatidylinositol (3,4,5)-trisphosphate (PIP3) molecule acts as a pivotal player in orchestrating cellular responses, serving as a key regulator for directional migration and cell polarization (3,4). The self-organized patterns of PIP3 waves observed in *Dictyostelium* cells have drawn attention for their relevance to similar processes in mammalian cells, and have been used as an alternative experimental model system for many human diseases (5,6,7,8).

PIP3 is a triply phosphorylated membrane lipid involved in the signaling pathway to recruit F-actin (9). It is generated from phosphatidylinositol (4,5)-bisphosphate (PIP2) by adding a phosphate group through phosphoinositide 3-kinase (PI3K), and then converted back by the phosphatase and tensin homolog (PTEN) (10). While PIP3 is correlated to other signaling activities, such as to Rac (11,12), it leads to activation of the mTOR signaling pathway and, hence, plays an important role in the cytoskeleton organization (13). Nanotopology-induced changes to the membrane of *Dictyostelium* cells trigger a pronounced rearrangement of the F-actin and PIP3 wave patterns (14,15). No F-actin wave patterns are observed upon latrunculin A treatment (16). However, even in the absence of F-actin, local membrane curvature pins and guides the PIP3 waves locally (17). The size and shape of the cell dictate the PIP3 wave dynamics on the membrane. Smaller cells lead to transient spot waves and larger ones to rotating waves (18). Only certain unstable modes with a wavelength limited by the cell size can be realized, as the size of the cell dictates the dynamics trough the excitable dispersion and restitution properties of the cell (19).

The confinement of the membrane, i.e., finite size effect, has been experimentally observed in the PAR cell polarity network of *C. elegans*, where a decrease in the cell size of the embryos destabilizes polarity and induces premature loss of division asymmetry (20). That is because the volume of a sphere increases faster than its surface area, as the volume scales with the cell radius *R* as $R^{3}$, whereas the cell surface scales as $R^{2}$. Dynamics on the membrane have been studied also from the theoretical and general biophysical point of view to explain biological systems, as cell membranes are active deformable surfaces (21,22,23,24,25). In these studies, the curvature of the membrane plays an important role to describe the self-organization of signals and membrane deformation in various biological systems, as the additional effect of curvature on the diffusion increases the complexity to understand and predict wave dynamics (26,27).

Also in *Dictyostelium* cells, membrane curvature plays an essential role for the guidance of cell migration. In curved nanotopological environments, it has been shown that the membrane curvature affects the wave formation of PIP3 and F-actin (14,15). This means that the cells actively sense and react to physical cues of the environment. The results of Yang et al. (15) indicate that the nanotopography is sensed directly by the cytoskeletal excitable network, whereas the signal-transduction excitable network is only indirectly affected due to feedback dynamics between both networks. On the contrary, we have shown that membrane curvature alone affects the self-organization of the PIP3 waves even in the absence of the cytoskeletal excitable network by the analysis of three-dimensional (3D) actin-polymerization inhibited *Dictyostelium* cell membranes (17). This is explained by the combination of molecular crowding and membrane curvature-limited diffusion, which means that PIP3 waves are guided or even pinned as spiral waves depending on the strength of membrane curvature. The additional effect of the membrane confinement and topology leads to a pronounced PIP3 wave directionality along the equatorial plane of the cell (18).

In this study, we numerically investigate the difference of the self-organization of spatiotemporal PIP3 waves on actin-polymerization inhibited cells with and without curvature-limited diffusion on the membrane, to verify the PIP3 dynamics observed experimentally, and gain insights of the fundamental nature of PIP3 pattern formation in the absence of the cytoskeletal network. We use a stochastic reaction-diffusion system with enzymatic Michaelis-Menten-type reactions on a 3D membrane, which closely resembles actin-polymerization inhibited *Dictyostelium* cells. By variations in size and shape of the cells similar to that observed experimentally, we compare and quantify the restitution dynamics and PIP3 wave propagation direction on the membrane. We found a Hopf-like bifurcation for membrane topologies with uniform diffusion, and a reverse period-doubling bifurcation for membranes with curvature-limited diffusion at the contact line for intermediate-sized nonspherical cells. That means that either dominant longitudinal or transversal stable PIP3 wave dynamics were observed. Larger cells showed secondary PIP3 wave formations, which led to chaotic wave formations. In the latter part of this work, we compare our results with the experimentally quantified results of *Dictyostelium* cells that partly recover the complex dynamics. Based on these results, we discuss possible scenarios that may explain the similarity and discrepancy between the biological and numerical results.

## Methods

The stochastic model was implemented in C, and the mesh preparation and computational analysis of the computed dynamics in MATLAB (The MathWorks, Natick, MA). A detailed description of the mesh preparation and computational analysis was published before for the analysis of experimentally observed *Dictyostelium* cells (17,18) together with a basic version of the implemented code that is available at MATLAB Central File Exchange (28).

### Computational platform

The stochastic model was computed on a NEC cluster using 20 cores (Intel Xeon Gold 6138 with 2.00 GHz, Skylake, 92 GB memory). The server architecture was provided by the High-Performance Computing Center Stuttgart (Stuttgart, Germany). A single simulation of 5000 s lipid membrane dynamics took about 24 h of simulation time at a single core.

### Membrane topology and mapping

The four spherical membrane topologies with $r_{A}$ between 0 and 30% were generated using a Delaunay triangulation routine (29), where $r_{A}$ is defined as the fraction of adhered membrane area $A_{\text{adh}}$ and nonadhered membrane area $A_{\text{non}-\text{adh}}$, as

(Equation 1) $$r_{A}=A_{\text{adh}}/A_{\text{non}-\text{adh}}\text{.}$$

The number of nodes for the four topologies was kept between about 3500 and 4000 nodes to ensure accurate mesh generation. Lists of the spatial position of the nodes and meshes, the index of the meshes, and their neighboring meshes, as well as the boundary length between meshes, were stored and provided to the stochastic model.

The meshes were visualized using the Mollweide projection, a homolographic equal-area projection, i.e., the area accuracy-based projection (30). The spherical coordinates (φ,θ) were transformed to cartesian coordinates (x,y), as follows:

(Equation 2) $$x=\frac{2\sqrt{2}}{\pi }\theta \;\cos\;\gamma$$

(Equation 3) $$y=\sqrt{2}\sin\;\gamma \text{.}$$

The auxiliary angle *γ* is given as

(Equation 4) 2γ+sin2γ=πsinφ,

and iteratively solved by the Newton-Raphson method (30,31).

### Gillespie algorithm (*τ*-leaping)

The reaction-diffusion system was solved using the stochastic Gillespie algorithm that calculates the process in time, executing the reactions in a period *τ*, instead of computing the time to every reaction (32,33). The state change for each molecular species *x* of the *n*th node is calculated, as

(Equation 5) $$x_{n}\left(t+\tau \right)=x_{n}\left(t\right)+\sum_{j}P\left(a_{j}\left(x\right)\right)+\sum_{m}D_{nm}\text{.}$$

P is the Poison distribution that depends on the kinetics, as

(Equation 6) $$a_{j}=A_{n}\tau R_{j}\left(x\right)\text{,}$$

where $A_{n}$ is the area of the node and $R_{j}$ are the reaction rates (propensity functions). The diffusion term $D_{nm}$ is calculated between neighboring nodes, as

(Equation 7) $$D_{nm}=\tau D_{n}A_{n}d_{nm}^{-1}s_{nm}H\left(s_{nm}\right)\text{,}$$

where $D_{n}$ is the diffusion constant of the *n*th mesh node, H is the Heaviside step function, $d_{nm}$ the distance between the three neighboring nodes *m*, and

(Equation 8) $$s_{nm}=\frac{x_{n}\left(t\right)}{A_{n}}-\frac{x_{m}\left(t\right)}{A_{m}}\text{.}$$

The neighboring nodes are updated accordingly, i.e., the number of molecular species are conserved during diffusion. In case of an unrealistic update of the molecular species, i.e., negative number of molecules in the node, the calculation was repeated.

### Tracking of signaling

For tracking the PIP3 lipid domains, spherical harmonic analysis was applied to smoothen the computed intensity distributions $I_{\text{mem}}\left(\varphi ,\theta \right)$ on the membrane, which enabled peek detection. The smoothed intensity distribution ${\tilde{I}}_{\text{mem}}\left(\varphi ,\theta \right)$ was computed up to the scalar harmonic degree l=3 (octupole moment) for each time step, as follows

(Equation 9) $${\tilde{I}}_{\text{mem}}\left(\varphi ,\theta \right)=\sum_{l=0}^{l=3}\sum_{m=-l}^{+l}f_{l}^{m}{\hat{Y}}_{l}^{m}\left(\varphi ,\theta \right)\text{,}$$

where the spherical harmonics is given by

(Equation 10) $$Y_{l}^{m}\left(\varphi ,\theta \right)=\sqrt{\frac{2l+1}{4\pi }\frac{\left(l-m\right)!}{\left(l+m\right)!}}P_{l}^{m}\left(\cos\;\theta \right)e^{im\varphi }$$

with the Legendre polynomials $P_{l}^{m}$ and ${\hat{Y}}_{l}^{m}$ the complex conjugated of $Y_{l}^{m}$. The spherical harmonic coefficients are defined as

(Equation 11) $$f_{l}^{m}=\sum_{\varphi }\sum_{\theta }I_{\text{mem}}\left(\varphi ,\theta \right)Y_{l}^{m}\left(\varphi ,\theta \right)\text{,}$$

where *φ* and *θ* denote the discrete and isotropically distributed node positions of the mesh. The parameter *m* is the order of the spherical harmonics and defined by *l*, as m=[−l,…,0,…,+l].

The velocity and the angular velocity components of the tracked domains were calculated as

(Equation 12) $$v=\sqrt{{\dot{r}}^{2}+v_{\theta }^{2}+v_{\varphi }^{2}}\text{,}$$

where the angular velocity components are

(Equation 13) $$v_{\varphi }=r\;\sin\;\theta \dot{\varphi }$$

(Equation 14) $$v_{\theta }=r\dot{\theta }\text{.}$$

## Results

### Self-organized lipid signaling in membranes

Phosphatidylinositol lipid dynamics have been modeled before on the basis of experimental observations of *Dictyostelium* cells (3). A simple reaction-diffusion system has been introduced that captures the spontaneous phosphatidylinositol lipid dynamics on a 1D ring, resembling the cell membrane periphery. Based on that model an improved reaction-diffusion system has been introduced with enzymatic Michaelis-Menten-type reactions (34). In that model, the reactions between PIP2 and PIP3 are not isolated, and the total concentrations of both lipids can change with time independently of PI3K and PTEN. The reaction scheme is given as

(Equation 15) $$\overset{q}{\to }\text{PIP}2\text{,}$$

(Equation 16) $$\text{PIP}2\overset{V_{\text{PI}3K}\frac{\left[\text{PIP}2\right]}{K_{\text{PI}3K}+\left[\text{PIP}2\right]}}{\to }\text{PIP}3\text{,}$$

(Equation 17) $$\text{PIP}3\overset{V_{\text{PTEN}}\left[\text{PTEN}\right]\frac{\left[\text{PIP}3\right]}{K_{\text{PTEN}}+\left[\text{PIP}3\right]}}{\to }\text{PIP}2\text{,}$$

(Equation 18) $$\text{PIP}2\overset{\lambda_{\text{PIP}2}\left[\text{PIP}2\right]}{\to }\emptyset \text{,}$$

(Equation 19) $$\text{PIP}3\overset{\lambda_{\text{PIP}3}\left[\text{PIP}3\right]}{\to }\emptyset \text{,}$$

(Equation 20) $${\text{PTEN}}_{\text{mem}}\overset{\lambda_{\text{PTEN}}\left[{\text{PTEN}}_{\text{mem}}\right]}{\to }{\text{PTEN}}_{\text{cyt}}\text{,}$$

(Equation 21) $${\text{PTEN}}_{\text{cyt}}\overset{V_{ass}\left[{\text{PTEN}}_{\text{cyt}}\right]\Lambda }{\to }{\text{PTEN}}_{\text{mem}}$$

with

$$\Lambda =\frac{K_{\text{PIP}3}}{K_{\text{PIP}3}+\left[\text{PIP}3\right]}\frac{\left[\text{PIP}2\right]}{K_{\text{PIP}2}+\left[\text{PIP}2\right]}$$

and

(Equation 22) $$\left[{\text{PTEN}}_{\text{cyt}}\right]=\left[{\text{PTEN}}_{\text{tot}}\right]-\chi \bar{\left[\text{PTEN}\right]}$$

where [PIP2], [PIP3], and [PTEN] are the membrane concentrations of PIP2, PIP3, and PTEN, respectively, and *q*, *χ*, $V_{i}$, $K_{i}$, and $\lambda_{i}$ are reaction constants (see Table S1). $\left[{\text{PTEN}}_{\text{cyt}}\right]$, $\left[{\text{PTEN}}_{\text{tot}}\right]$, and $\bar{\left[\text{PTEN}\right]}$ are the cytosolic, total and average membrane concentration of PTEN.

**Figure 1:**
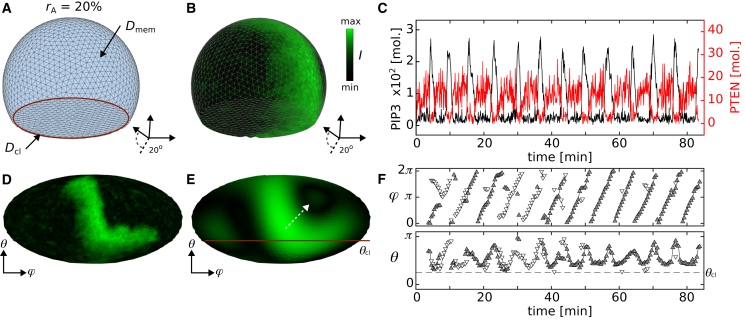
Mesh and signal visualization. (*A* and *B*) Show the 3D views of a plain mesh and a mesh with mapped computed PIP3 intensity signaling. The examples show meshes with $r_{A}=20\%$ from the bottom with an inclination angle of $20^{\circ }$. (*C*) Shows the molecular changes over time of PIP3 (*black*) and PTEN (*red*) at a single grid. (*D* and *E*) Show the 2D Mollweide projection of the example shown in (*B*), where $\theta_{\text{cl}}$ defines the contact line between adhesive and nonadhesive part of the membrane that is indicated by the red solid line in (*A*) and (*E*). (*E*) Shows the smoothed intensity distribution obtained by spherical harmonics analysis of the simulated data shown in (*B*) and (*D*). The white dashed arrow indicates the direction of wave propagation from the detected peak. The color bar indicates the normalized PIP3 signaling intensity. (*F*) Shows the spatiotemporal position of the detected wave positions in spherical coordinates. The dashed line indicates the contact line. To see this figure in color, go online.

In this study, these reaction-diffusion processes are computed on 3D cell-shaped surfaces using the *τ*-leaping Gillespie algorithm-based method (32,33). The surfaces resemble actin-polymerization inhibited *Dictyostelium* cells (18), which are generated by a Delaunay triangulation routine (see methods). The diffusion is considered for PIP2 and PIP3 as a stochastic jump process between neighboring grids and the reaction rates (propensity functions on top of the arrows of (Equation 15), (Equation 16), (Equation 17), (Equation 18), (Equation 19), (Equation 20), (Equation 21) with a discrete time stepping of $\tau =2\times 10^{-4}$ s. The dynamics were simulated for a duration of $5\times 10^{3}$ s for different cell radii ranging from R= 5 to 16 *μ*m. Additional variations of the membrane topology between $r_{A}=$ 0% (spherical) and 30% (flattened) led to 44 different cell topologies. Fig. 1 *A* shows the grid of a cell with $r_{A}=$ 20%. The contact line between substrate adhered membrane (flat surface) and nonadhered membrane is highlighted by a red line. The diffusion on the membrane surface is considered as $D_{\text{mem}}=0.2$ *μ*m^2^/s equally for PIP2 and PIP3 (35). The diffusion at the contact line is considered either as $D_{\text{mem}}=D_{\text{cl}}$ or $D_{\text{mem}}\neq D_{\text{cl}}=0.04$ *μ*m^2^/s (see Fig. S1). We refer from here on to membranes with uniform diffusion and diffusion-limited membranes, respectively.

Fig. 1*B* shows a snapshot of a typical PIP3 domain distribution on the surface of a cell, and Fig. 1 *C* shows the temporal dynamics of PIP3 (*black*) and PTEN (*red*) at a single membrane node. To track these PIP3 domains on the membrane over time, spherical harmonics analysis was applied and higher-order modes were removed. This simplifies the complex patterns on the surface and enables wave detection. The same approach was applied experimentally at *Dictyostelium* cells before (18). The 2D projections (see methods) of that cell are shown in Fig. 1, *D* and *E*. The unprocessed simulated and the by spherical harmonics simplified PIP3 distributions are shown. The position and direction of the PIP3 domain dynamic are indicated by the white dashed arrow. The horizontal line indicates the contact line, similarly as indicated in Fig. 1 *A*. The area below that line indicates the substrate adhered part of the membrane. Up to two domains at a time were tracked on the surface of the membrane. The spatial position of the waves is tracked by spherical coordinates with the azimuthal angle *φ* (latitude) and the polar angle *θ* (longitude). Fig. 1 *F* shows an example. Up to two domains are shown with white and gray triangles at a time. When one PIP3 wave is present (e.g., *white*), the secondary wave is shown by the opposite color (e.g., *gray*). Same waves are connected by a black line.

### Membrane topology and diffusivity

**Figure 2:**
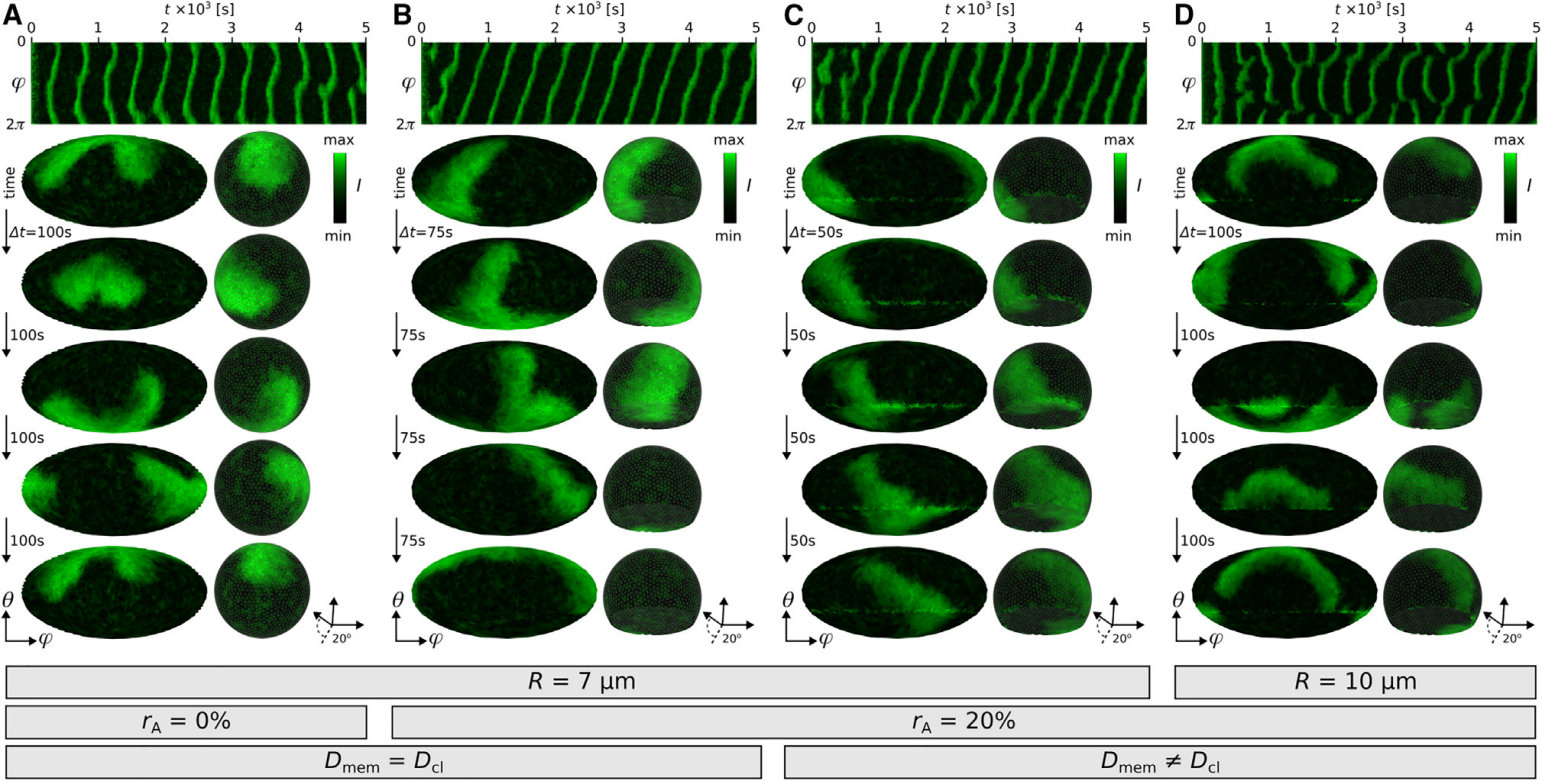
Influence of membrane shape and curvature-limited diffusion on PIP3 wave dynamics. (*A–D*) Show typical examples with different membrane properties (*R*, $r_{A}$, and *D*). The left and middle panels are five snapshots—2D map and 3D view—of the PIP3 membrane distribution. The right panels show the kymographs obtained at the equatorial section of the cell membrane (θ=0). (*A* and *B*) Show cells with constant diffusion at the entire membrane but different cell shape, (*B* and *C*) show cells of same shape and geometry but different diffusion properties, and (*C* and *D*) show cells with curvature-limited diffusion at the contact line but different cell size. The 3D views show the ventral membrane from the bottom with an inclination angle of $20^{\circ }$. The color bars indicate the normalized PIP3 signaling intensity. To see this figure in color, go online.

Depending on the membrane topology various dynamics can be observed (Fig. 2, *A–D*). Perfectly spherical cells ($r_{A}=$ 0%) do not have a symmetry axis, so stable rotating waves can be observed in various configurations leading to horizontally and vertically propagating waves. An example of the latter is shown in Fig. 2 *A*. Membrane snapshots show one period of two rotating spiral waves. Their tips, i.e., phase singularities, are located on the opposite side of the cells with a common wave front that travels vertically up and down. This dynamic is also indicated by the vertical lines in the Kymograph, which is taken at the equatorial height of the cell (θ=π/2). Adherent cells ($r_{A}>$ 0%), as shown exemplarily in Fig. 2 *B* ($r_{A}=$ 20%), have the tendency to exhibit stable vertically rotating waves. The spiral tips are located at the close proximity to the top and the bottom of the cell membrane due to the vertical symmetry axis of the membrane. This is because excitable waves tend to propagate along the longest path on spatially confined membrane surfaces due to their inhibitory signaling properties. In other words, the wave front propagates the path where the excitable PIP3 wave did not occur for the longest time, as the PIP3 wave propagates in its own inhibitory tail. In adherent cells those are the great circles in close proximity to the equatorial line.

**Figure 3:**
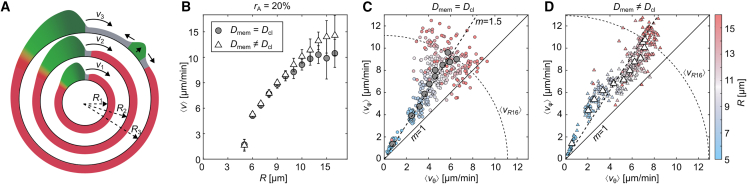
Influence of curvature and spatially limited diffusion on PIP3 waves in cells with $r_{A}=20\%$. (*A*) Shows a scheme on the effect of restitution and dispersion in three 1D rings of different radius, where $v_{2}\left(R_{2}\right)>v_{1}\left(R_{1}\right)$ and $v_{3}\left(R_{3}\right)>v_{2}\left(R_{2}\right)$. The average speed $\left\langle v_{R_{3}}\right\rangle$ of all waves may be smaller or larger than $v_{2}$ depending on the spatiotemporal dynamics of secondary waves (upper right in $R_{3}$). The colors indicate the excited (*green*), inhibitory (*red*), and rested states (*gray*). (*B*) Shows the average of the mean velocities ⟨v⟩ depending on the cell radius *R* (restitution). (*C* and *D*) Show the relation between the mean velocity components of the transversal $\left\langle v_{\theta }\right\rangle$ and longitudinal $\left\langle v_{\varphi }\right\rangle$ directions for each simulation depending on *R* and the diffusion properties, respectively. The average values—sorted by radius—are shown by large gray circles (*C*) and large white triangles (*D*). The dashed line shown in (*C*) is the linear fit of the average values (*large gray circles*) with a slope of m=1.5, and is plotted for comparison in (*D*) too. The dashed arc depicts the theoretical average of the mean velocities $\left\langle v_{R16}\right\rangle$ at R=16*μ*m. To see this figure in color, go online.

When introducing diffusion-limited PIP3 signaling at the contact line of the membrane ($D_{\text{cl}}=0.04$ *μ*m^2^/s), which more closely relates to the experimentally observed actin-polymerization inhibited (17) and motile *Dictyostelium* cells (14), stable horizontally rotating waves are observed (Fig. 2 *C*) too. However, no cases of vertically rotating waves are observed, contrary to cells with uniform diffusibility ($D_{\text{mem}}=D_{\text{cl}}$) where also cases of stable vertically rotating waves are observed. Thus, despite the same cell topology (rA= 20%, R= 7 *μ*m), the diffusion limitation at the contact line leads to the stabilization of the PIP3 wave directionality. In larger cells, more complex PIP3 wave dynamics are observed. Fig. 2 *D* shows an R= 10 *μ*m sized cell, where the strong horizontal directionality is broken, as a secondary PIP3 wave appears. This is possible, as there is a natural speed limit defined by the dispersion properties of the system, i.e., relationship between wave speed and rotational frequency. The wave needs more time to propagate around the membrane with the increase in cell size, which in turn enables the formation of a secondary PIP3 domain if the cell is sufficiently large. Fig. 3 *A* depicts a simplified 1D scheme for three different radii. The larger the radius of the 1D circle, the larger the wave speed. The wave speed saturates at a certain radius *R*, here $R_{2}<R<R_{3}$, and leads to longer delays within the wave front and inhibitory wave tail, where the formation of secondary waves may occur. In the case of diffusion-limited membranes, these secondary waves appear more prominently on the contact line (Fig. 2 *D*, *third snapshot*), contrary to membranes with uniform diffusibility, where those waves do not appear at any specific membrane location.

### Membrane curvature restricted diffusion

Fig. 3*B* statistically compares the dispersion relationships between PIP3 waves on membranes ($r_{A}=$ 20%) with uniform and contact line limited diffusibility as gray circles and white triangles. The cell radius *R* defines the mean velocity ⟨v⟩ for both cases fairly well. For larger cells, a dispersion of ⟨v⟩ is observed, as secondary PIP3 waves lead to more complex wave-wave interactions. Although the dispersion relationships are very similar, there is a profound difference for the mean velocity components. Fig. 3, *C* and *D* show the relations between the transversal $\left\langle v_{\theta }\right\rangle$ and longitudinal $\left\langle v_{\varphi }\right\rangle$ velocities of those waves for each simulation. In case of uniform diffusibility, the average velocity ratio (*gray circles*) for each cell size is constant at a slope of about m=1.5 (*dashed line*). With the increase in cell size, an increase of the dispersion of possible velocity ratios is observed, ranging from unity m=1 up to a ratio of about m=3 (see *small magenta circles*). On the contrary, PIP3 waves on membranes with contact line limited diffusibility have a slope beyond m=1.5 for smaller cells and a slope beneath m=1.5 for larger cells (Fig. 3 *C*). Only at R= 9 *μ*m is a comparable aspect ratio (m=1.5) observed. Also the increase in dispersion for larger cells is visibly reduced compared with the case of uniform diffusibility. The lower velocity ratio and lower velocity dispersion for larger cells can be explained by the pinned PIP3 wave tips (singularities) on the contact line that further enables and stabilized the formation of the secondary waves on the contact line between the two wave tips (see also Fig. 2 *D*). PIP3 waves in smaller cells are slowed down by their inhibitory wave tail, but stabilized by the cell asymmetry and guided along the contact line, which leads to an increased velocity ratio (m>1.5).

### Bifurcation of lipid dynamics

**Figure 4:**
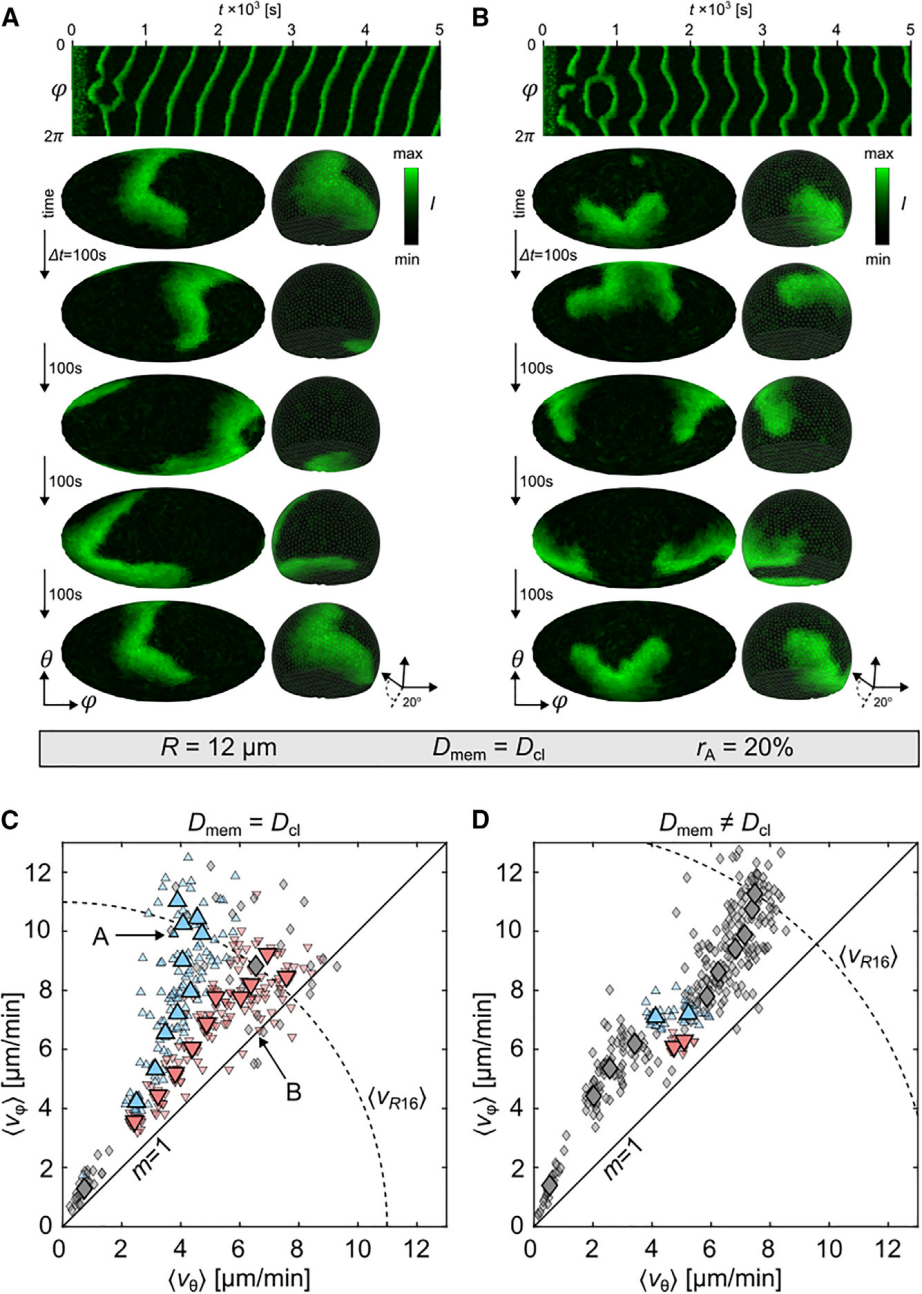
Longitudinal and transversal stable domain dynamics in cells with $r_{A}=20\%$. (*A*) Shows longitudinal PIP3 wave dynamics with stable pinning sites on the bottom and top of the membrane. (*B*) Shows transversal PIP3 wave dynamics with stable pinning sites around the equator of the membrane. Shape, size, and diffusion are the same for both cells. The 3D views show the ventral membrane from the bottom with an inclination angle of $20^{\circ }$. (*C* and *D*) Show the *k*-means analysis sorted data for the diffusion properties $D_{\text{mem}}=D_{\text{cl}}$ and $D_{\text{mem}}\neq D_{\text{cl}}$, respectively. Shown are the relation between the mean velocity components of the transversal $\left\langle v_{\theta }\right\rangle$ and longitudinal $\left\langle v_{\varphi }\right\rangle$ directions for each simulation depending on *R*. Blue upward- and red downward-pointing triangles illustrate the upper and lower branch of the bifurcated data. Data shown with diamonds are not analyzed by *k*-means. The solid black line indicates the slope m=1. The dashed arc depicts the theoretical average of the mean velocities $\left\langle v_{R16}\right\rangle$ at R=16*μ*m. The illustrated wave dynamics shown in (*A*) and (*B*) are indicated in (*C*) by arrows. To see this figure in color, go online.

For larger cells, PIP3 domains have the tendency to stabilize their propagation directionality either in longitudinal or transversal direction. Fig. 4, *A* and *B* show two example cells with R=12 *μ*m, $D_{\text{mem}}=D_{\text{cl}}$, and $r_{A}=20\%$. The propagation direction is stable once a certain propagation direction is set after about $1\times 10^{3}$ s. For cells with diffusion-limited membranes, this tendency gets broken and mainly transversal propagation is observed, as the PIP3 wave tips get pinned on the contact line, thus forcing the PIP3 domains to an transversal directionality (Fig. 3 *D*). This enforced pattern stabilization, can be explained by the bifurcation theory. So we hypothesize two stable solutions and quantify the velocity tuples $\left\langle v_{\theta }\right\rangle$ and $\left\langle v_{\varphi }\right\rangle$ by a *k*-means unsupervised, clustering algorithm with either two solutions k=2 when a significant difference between the calculated clusters is observed, or one solution k=1 (no bifurcation). Fig. 4 *C* shows the result for the case of uniform diffusion on cells with $r_{A}=20\%$. The blue up- and red down-pointing triangles define the two identified clusters for cells with the same cell radius. The gray diamonds depict the cases where no bifurcation was identified, which was for either the smallest (R=5 *μ*m) or the largest simulated radius R=16 *μ*m. Cells with the largest radius show almost no cases of prominent longitudinal domain direction. Thus, PIP3 domain dynamics are observed within the lower branch that shows more balanced domain propagation between $\left\langle v_{\theta }\right\rangle$ and $\left\langle v_{\varphi }\right\rangle$ (*red down-pointing triangles*). The larger symbols depict the average position of the identified clusters. Similarly, we analyzed the results for diffusion-limited membranes (Fig. 4 *D*). Here, only at the point of the slope transition (m≈1.5, see Fig. 3, *C* and *D*) for radii R=9 *μ*m and R=10 *μ*m is a bifurcation identified.

**Figure 5:**
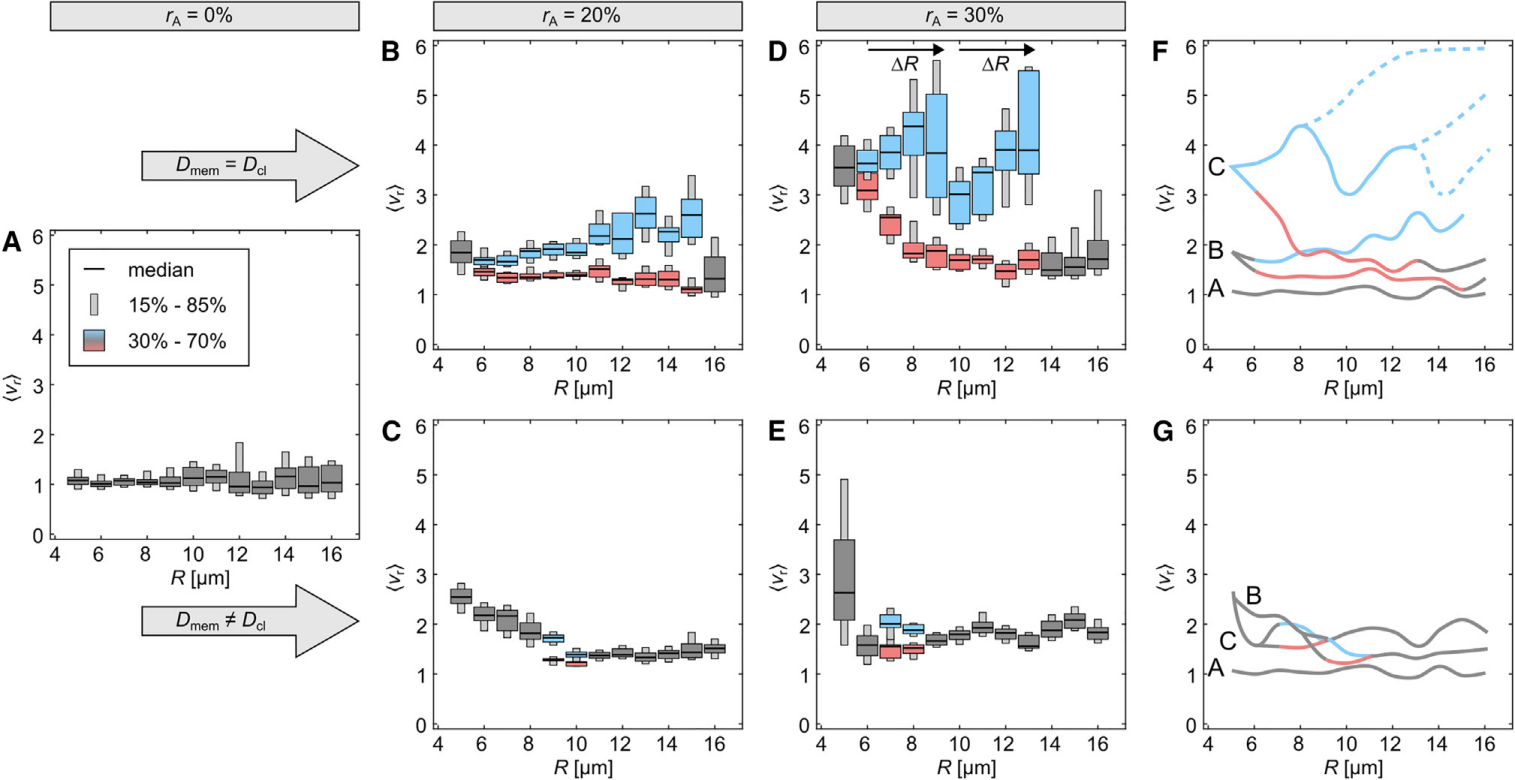
Bifurcation of the wave velocity ratio in confined membranes. (*A*–*E*) Show whisker plots of the mean velocity ratio $\left\langle v_{r}\right\rangle$ depending on the shape ($r_{A}$), size (*R*), and diffusion properties of the membranes. The blue upper and red lower whiskers correspond to the *k*-means analysis sorted relation between the mean velocity components of the transversal $\left\langle v_{\theta }\right\rangle$ and longitudinal $\left\langle v_{\varphi }\right\rangle$ directions (see Fig. 4, *C* and *D*, for comparison). Whiskers shown with gray color are data that were not analyzed by *k*-means. (*F* and *G*) Show for comparison the smoothed median approximation of the data with uniform diffusion ($D_{\text{mem}}=D_{\text{cl}}$) and membrane curvature reduced diffusion ($D_{\text{mem}}\neq D_{\text{cl}}$). Prolonged dashed lines indicate extension of the unstable hypothetically drawn bifurcation branching. To see this figure in color, go online.

For proof of our bifurcation hypothesis without loss of generality, we additionally performed simulations for cells with $r_{A}=0\%$ and $r_{A}=30\%$, applied the same *k*-means analysis, and considered the mean velocity ratio

(Equation 23) $$\left\langle v_{r}\right\rangle =\left\langle v_{\varphi }/v_{\theta }\right\rangle$$

as a function of the cell radius *R*. This should not be confused with the ratio of the mean velocity components $\left\langle v_{\theta }\right\rangle /\left\langle v_{\varphi }\right\rangle$. Fig. 5 shows the comparison between uniform diffusion and diffusion-limited membranes. The color of the whisker boxes depicts the clusters, as shown in Fig. 4, *C* and *D*. Gray depicts no bifurcation (k=1) and blue and red define the two clusters of the typical domain propagation patterns. Fig. 5 *A* shows the case of spherical cells (see also Fig. 2 *A*). The median of all obtained $\left\langle v_{r}\right\rangle$ is about unity, independent of the cell radius, as there is neither a topological asymmetry in the cell shape nor any other membrane property that may lead to a statistically reoccurring symmetry break in the pattern formation on the confined cell membranes. Fig. 2 *B–E* shows $\left\langle v_{r}\right\rangle$ for the combination of nonspherical cells ($r_{A}=20\%$ and $r_{A}=30\%$) and different membrane diffusion properties ($D_{\text{mem}}=D_{\text{cl}}$ and $D_{\text{mem}}\neq D_{\text{cl}}$). Generally, small cells (R=5 *μ*m) show no bifurcation. With the increase in cell size $\left\langle v_{r}\right\rangle$ decreases and eventually saturates at a low value with almost no cases of larger $\left\langle v_{r}\right\rangle$, i.e., dominant longitudinal velocity $\left\langle v_{\varphi }\right\rangle$. The second branch (*blue data*) shows independently of the cell topology an initial increase in $\left\langle v_{r}\right\rangle$ when $D_{\text{mem}}=D_{\text{cl}}$. With an increase in *R*, $\left\langle v_{r}\right\rangle$ destabilizes until no such patterns are observed anymore. Especially for the case of $r_{A}=30\%$ (Fig. 5 *D*), a sudden drop in $\left\langle v_{r}\right\rangle$ at R=10 *μ*m is observed, which recovers to larger $\left\langle v_{r}\right\rangle$ until the disappearance of those patterns at R=14 *μ*m. This can be explained by an additional bifurcation with unstable solutions, as indicated by the scheme shown in Fig. 5 *F*. The stable solutions are indicated by solid lines in the respective color. The assumed unstable solutions are indicated as blue dashed lines. It is one possible explanation, although this is a purely hypothetical solution, which we cannot back up by data. The periodic increase of the second branch (*blue data*) repeats at about ΔR=4 *μ*m, indicating a relationship between dispersion properties of the PIP3 domain dynamics and the confined membrane topology. One might identify a third period for R>13 *μ*m due to the occurrence of a few cases that fall in that region, which is indicated by the elongated gray whisker boxes (see also Fig. S3 *A*). However, we refrain from defining a third period as we cannot back up the data statistically. For the same reasons we also do not define a third lower branch for a few simulations that resulted in $\left\langle v_{r}\right\rangle \approx 1$ (Fig. S3 *A*). The diffusion-limited membranes show a different branching type (Fig. 5, *C* and *E*). A reverse period-doubling bifurcation (antimonotonicity) in $\left\langle v_{r}\right\rangle$ is observed (36,37). In both cases the bifurcation is less prominent, as it only ranges for about ΔR=2 *μ*m and shows minor variations in $\left\langle v_{r}\right\rangle$. Arguably, differences in $\left\langle v_{r}\right\rangle$ are weak compared with the case of $D_{\text{mem}}=D_{\text{cl}}$; thus, they may not be bifurcations after all. However, we refer to Figs. 4 *D* and S3 *B*, where a clear difference in $\left\langle v_{\varphi }\right\rangle$ between the two stable solutions is observed. We leave it up to the reader and future investigations to argue about this findings.

### Experimental comparison

**Figure 6:**
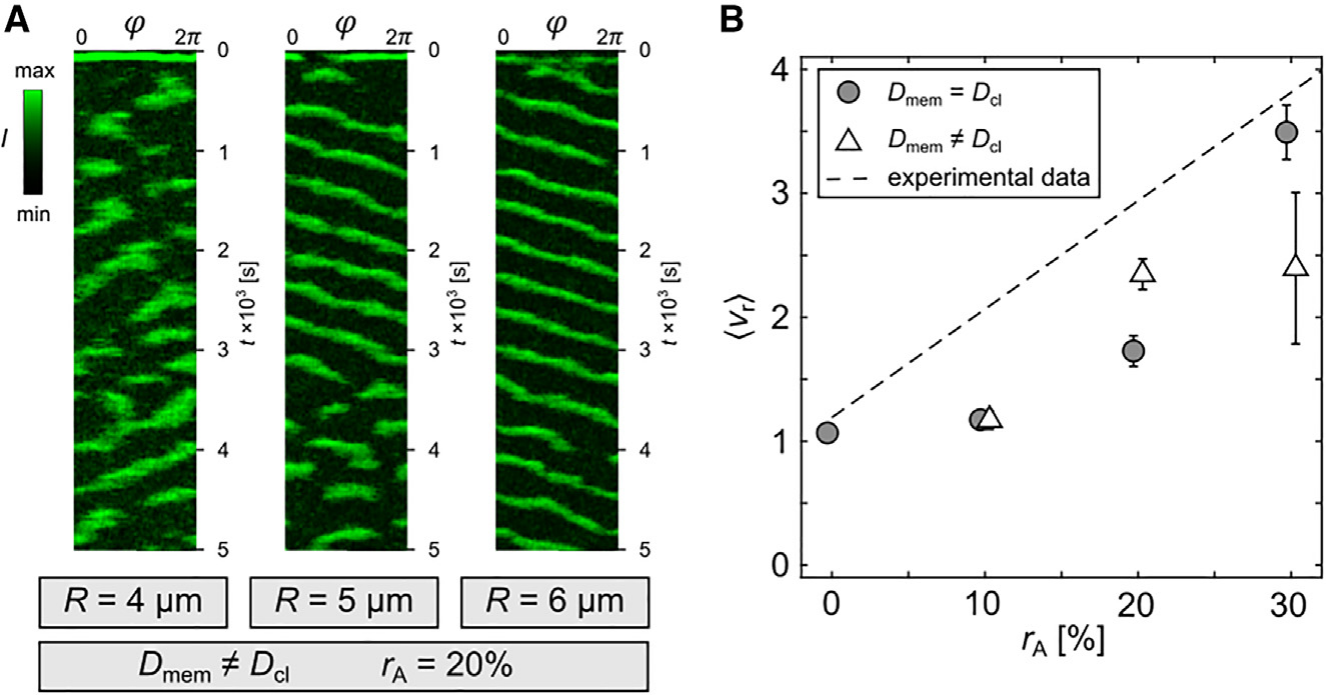
Comparison with experimental data of *Dictyostelium* cells. (*A*) Shows three kymographs of simulations with different cell radii: chaotic (*left*, R=4*μ*m), semistable (*middle*, R=5*μ*m), and stable (*right*, R=6*μ*m) PIP3 domain dynamics ($D_{\text{mem}}\neq D_{\text{cl}}$, $r_{A}=20\%$). (*B*) Shows the experimentally obtained (Hörning et al. (17)) analytical relation between $r_{A}$ and $\left\langle v_{r}\right\rangle$ with the dashed line. The gray circles and white triangles show the comparison with the simulations with $D_{\text{mem}}=D_{\text{cl}}$ and $D_{\text{mem}}\neq D_{\text{cl}}$ for realistic *Dictyostelium* cell sizes of R=5 and 6 *μ*m only. To see this figure in color, go online.

Latrunculin A-treated *Dictyostelium* cells have a typical size of about R=5 *μ*m to R=6 *μ*m. The inhibition of actin-polymerization leads to a rounding up of the cell membrane with an adhesive membrane ratio of up to about 30% (18), which is regulated by the cortical tension of the cell (38,39). Periodically transversal and longitudinal traveling PIP3 domains, as well as, spatially localized temporal PIP3 domains, are observed depending on the size, shape, and phenotype of the *Dictyostelium* cells (18). In numerical simulations, similar pattern formation can be observed. While larger cells exhibit traveling wave-like domains (Fig. 2), smaller cells exhibit more chaotic unstable domain dynamics (Fig. 6 *A*). The interactions of the domains with their own inhibitory tails are stronger in smaller cells due to the limited membrane size and their confinement. Thus, the smaller the cell, the more unstable the domain dynamics.

To validate the experimentally suggested membrane curvature influenced diffusion in *Dictyostelium* (14,17), we compare the experimentally quantified relation between rA and $\left\langle v_{r}\right\rangle$ in Hörning et al. (17) with the numerical results of this study (Fig. 6 *B*). In the case of uniform diffusion ($D_{\text{mem}}=D_{\text{cl}}$, *gray circles*), perfectly round ($r_{A}=0\%$) and strongly adhered ($r_{A}=30\%$) cells are in good agreement with the experimental relation. However, simulations of the intermediate cell topologies ($r_{A}=10\%$ and $r_{A}=20\%$) do not agree well. The simulations underestimate the experimental data and, thus, indicate a missing cell property or dynamic in the model. In the case of membrane curvature reduced diffusion ($D_{\text{mem}}\neq D_{\text{cl}}$), the domain guiding effect of the contact line significantly increases $\left\langle v_{r}\right\rangle$ for $r_{A}=20\%$, whereas there is almost no measurable effect for cells with $r_{A}=10\%$. Cells with $r_{A}=30\%$ show more unstable solutions (as shown in Fig. 6 *A*), and therefore lead to a broader distribution of possible $\left\langle v_{r}\right\rangle$ (see also Fig. 5 *E*, R=5 *μ*m).

The results indicate that the model that considers membrane curvature reduced diffusion represents more closely the experimental data. Cells with larger adhesion ratio ($r_{A}=30\%$) follow an opposite trend. This is due to the fact that the propagation speed is close to the critical point where stable waves can form (see Fig. S2 *C*, R=5 and 6 *μ*m). Furthermore, they are barely observed and, therefore, do not account for the majority of experimentally observed cells (17,18). Despite the improvements to the model, which more realistically represent the experimental findings compared with uniform membrane diffusion, there is still something missing that remains to be elucidated in experiments. A possible explanation might be linked to the diffusion properties of the crowded and structural cytosolic environment that can lead to heterogeneous intracellular diffusion (40). The current model does not account for that, but considers a simplified linear global PTEN uptake only (see Eq. 22). Another experimental finding that still needs to be understood is the vertical gradient of PIP3 signaling activity observed in *Dictyostelium* cells (17), which was obtained by spatial fluctuation analysis (41). The vertical quantification of the fluctuation parameter showed a lower value on top of the cell that increases toward the equatorial membrane with a constant slope in most cells. The cause of that general trend remains to be elucidated and, therefore, could not be considered in the current model.

## Conclusions

We investigated PIP3 domain dynamics with and without curvature-limited diffusion at the contact line of the cell membrane in the first part of this study. Without loss of generality, we considered larger cells up to R=16 *μ*m, as those dynamics might be observed in other cell types. We found a Hopf-like bifurcation for the observed PIP3 dynamics in the case of uniform membrane diffusion independently of the topology of nonspherical cell membranes (Fig. 5, *B* and *D*). The domains propagate along either the longitudinal or transversal direction, which can be visualized with $v_{r}\left(R\right)$. In both cases, a pair of singularities (spiral wave cores) is observed. In the case of longitudinally directed domain propagation, one singularity propagates along the contact line and the other one freely at the top of the cell membrane (Fig. 4 *A*). On the contrary, for transversally directed domain propagation, both singularities are horizontally aligned and rotate as spiral waves with opposite directionality in the proximity of the equatorial height of the cell (Fig. 4 *B*). For larger cells, only the latter is observed, as secondary PIP3 domains may appear, forcing the domains into transversal direction. A reverse period-doubling bifurcation (antimonotonicity) is observed in $v_{r}\left(R\right)$ for membranes with curvature-limited diffusion at the contact line for intermediate-sized nonspherical cells (Fig. 5, *C* and *E*). This type of bifurcation is observed at the transition between the initially decreasing $v_{r}\left(R\right)$ and the in $v_{\left(r\right)}$ observed asymptotic limit for larger cells. Thus, PIP3 domains in smaller cells, i.e., before this transition, are strongly influenced by the contact line, and PIP3 domains in larger cells are mainly influenced by (1) the asymmetry of the membrane topology and (2) the contact line as the main source of secondary waves (Fig. 2 *D*). Because the influence of the contact line decreases significantly for large cells, $v_{r}$ becomes comparable for cells with and without curvature-limited diffusion (see Fig. S4 for R=16 *μ*m). The limit of $v_{r}$ depends on the strength of adhesion, which means the larger $r_{A}$ the larger $v_{r}$. Perfectly round cells ($r_{A}=0\%$) have a $v_{r}≃1$. On the contrary, fully adhered cells with an intact actin cytoskeleton, as cultured on glass-bottom dishes, have most likely a much larger $v_{r}$ than the case considered in this study, i.e., $v_{r}\gg 2$. This is because such cells have an estimated $r_{A}≃50\%$, as the surface-adhered membrane and the upper free membrane are of approximately the same size. Furthermore, it would be interesting to consider membrane topologies that model tissue embedded cells and cells that form macropinosomes (42).

In the latter part of this study, we compared our results with experimental findings observed on 3D *Dictyostelium* cell membranes (17). We restricted the comparison to cells with radii of R=5 and 6 *μ*m, as experimentally observed before (18). The introduced curvature-limited diffusion reflects more closely the experimental data for $r_{A}≃20\%$. On the contrary, for less adhered cells ($r_{A}≃10\%$) virtually no effect has been observed. The fraction of adhered membrane is too small, and hence does not affect $\left\langle v_{r}\right\rangle$. While the observed discrepancy between the experiments and numerical results might be mainly caused by the still unclear origin of the vertical gradient of PIP3 signaling activity on *Dictyostelium* cell membranes, as mentioned before, there are also other improvements to the model that can be made. The restitution and dispersion properties could be tuned to account for the biological variance in the dynamics, and the cytosolic dynamics more realistically modeled, including the effect of the intracellular crowding. The diffusion constant might differ for the adhered membranes, and the diffusion constant on the strongly curved membrane is arbitrarily chosen, because there are no experimental data available yet. Also the quantification of the PIP3 domain dynamics can be improved. Only lower scalar harmonics are considered for enabling PIP3 peak detection. Larger scalar harmonic degrees increase the accuracy of peak detection, but might induce errors due to unwanted peak jumps. In this paper, we investigate the effect of diffusion by considering the 1D curvature defined by the adhesion angle of the cell membrane only. However, a better measure might be the Gaussian curvature *K*, as it has been already applied to describe numerically the dynamics of the Belousov-Zhabotinsky reaction on nonuniformly curved surfaces (43). *K* is defined on the surface as the product of the two principle curvatures, as $K=\kappa_{1}\kappa_{2}$. On the contact line of symmetric actin-inhibited cell membranes, as considered in this study, $\kappa_{1}=\kappa_{\theta }\left(\theta_{\text{cl}}\right)$ and $\kappa_{2}=\kappa_{\varphi }=R_{\text{cl}}{\left(\varphi \right)}^{-1}$ with $R_{\text{cl}}$ as the horizontal radius along the contact line.

In future, the model might be also applied and compared with more complex biological processes and dynamics, such as to migrating cells (14,15) and macropinocytosis (42), by adding mechnochemical signaling pathways that control filamentous actin assembly (9).

## Author contributions

S.E. and M.H. implemented the model and analyzed the data. M.H. conceptualized and designed the research and wrote the manuscript. All authors contributed to the article and approved the submitted version.

## Supporting material

Document S1. Figures S1–S4 and Table S1

Document S2. Article plus supporting material
