# Supplementary material for: Self-organization of PIP3 waves is controlled by the topology and curvature of cell membranes
Source: Biophys J. 2024 Mar 21;123(9):1058–68. doi: 10.1016/j.bpj.2024.03.022 (PMC11079865; doi:10.1016/j.bpj.2024.03.022)
Supplement: Document S1. Figures S1–S4 and Table S1 [file mmc1.pdf]

Biophysical Journal, Volume *123*

Supplemental information

Self-organization of PIP3 waves is controlled by the topology and curvature of cell membranes

Sema Erisis and Marcel Hörning

Supporting Information

Self-organization of PIP3 waves is controlled by the topology and curvature of cell membranes.

Sema Erisis[1] and Marcel Hörning[1]

[1] *Institute of Biomaterials and Biomolecular Systems, University of Stuttgart, Stuttgart, Germany*

TABLE I. Reaction constants and initial concentrations of the model.

Parameter	value	dimension	short description
[PIP2]	700	d.l.	initial global PIP2 concentration
[PIP3]	5	d.l.	initial global PIP3 concentration
[PTEN]	2	d.l.	initial global PTEN concentration
$[\text{PTEN}]_{total}$	0.1	μM	total concentration of PTEN
q	45	molecules$\cdot \mu m^{-2} \cdot s^{-1}$	PTEN-independent PIP2 supply rate
V_{PI3K}	500	molecules$\cdot \mu m^{-2} \cdot s^{-1}$	max. velocity of PIP2 phosphorylation by PI3K
K_{PI3K}	3500	molecules$\cdot \mu m^{-2}$	Michaelis constant of PI3K phosphorylation reaction
V_{PTEN}	15	s^{-1}	dephosphoryl. rate of PIP3 by PTEN
K_{PTEN}	50	molecules$\cdot \mu m^{-2}$	Michaelis constant of PTEN phosphorylation reaction
λ_{PIP3}	0.2	s^{-1}	PTEN-independent PIP3 degradation rate
λ_{PIP2}	0.002	s^{-1}	PI3K-independent PIP2 degradation rate
λ_{PTEN}	1.0	s^{-1}	dissociation rate of PTEN from membrane
V_{ass}	1300	molecules$\cdot \mu m^{-2} \cdot \mu M^{-1} \cdot s^{-1}$	association rate of PTEN to membrane
K_{PIP2}	3000	molecules$\cdot \mu m^{-2}$	Michaelis constant of PIP2 for PTEN
			associated to membrane
K_{PIP3}	120	molecules$\cdot \mu m^{-2}$	half-maximum concentration of [PIP3]
χ	0.001	$\mu M \cdot \mu m^{-2} \cdot molecules^{-1}$	transform surface to volume concentration

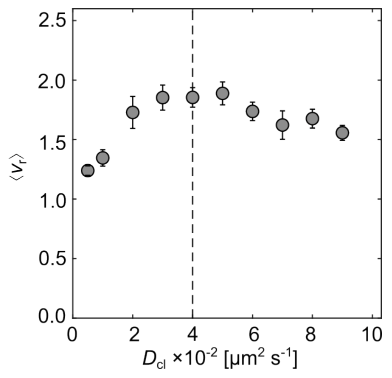

Fig. S1 Determination of the optimal diffusion on the contact line of the membrane. The optimal diffusion $D_{\text{cl}} = 0.04\ \mu\text{m}^2/\text{s}^{-1}$ (dashed line) was selected for cells with $R = 6\ \mu\text{m}$, $r_{\text{A}} = 20\%$ and $D_{\text{mem}} = 0.2\ \mu\text{m}^2/\text{s}^{-1}$. For each condition 20 simulation were computed, and the average $\langle v_{\text{r}} \rangle$ was calculated. The error bars indicate the standard error.

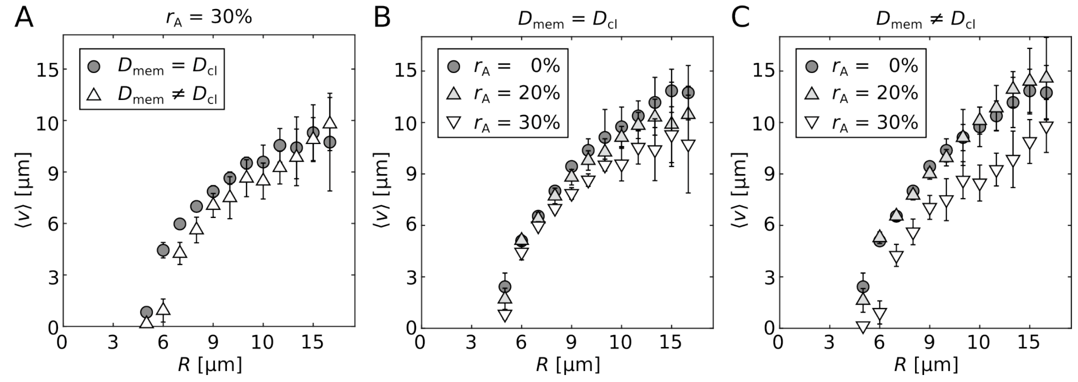

Fig. S2 Comparison of restitution curves. **A** shows the restitution curves, i.e. the average of the mean velocities $\langle v \rangle$ depending on the cell radius R for $r_{\text{A}} = 30\%$. **B** and **C** show the restitution curves of three cell shapes (r_{A}) for $D_{\text{mem}} = D_{\text{cl}}$ and $D_{\text{mem}} \neq D_{\text{cl}}$, respectively.

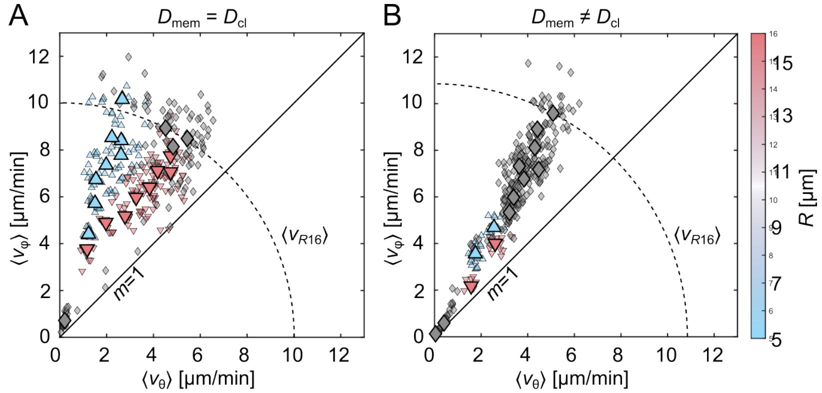

Fig. S3 Longitudinal and transversal stable domain dynamics in cells with $r_A = 30\%$. **A** and **B** show the by k-means analysis sorted data for the diffusion properties $D_{mem} = D_{cl}$ and $D_{mem} \neq D_{cl}$, respectively. Shown are the relation between the mean velocity components of the transversal $\langle v_\theta \rangle$ and longitudinal $\langle v_\varphi \rangle$ directions for each simulation depending on R. Blue upward- and red downward-pointing triangles illustrate the upper and lower branch of the bifurcated data. Data shown with diamonds are not analyzed by k-means. The solid line indicates the slope $m = 1$. The dashed arc depicts the theoretical average of the mean velocities $\langle v_{R16} \rangle$ at $R = 16$ µm.

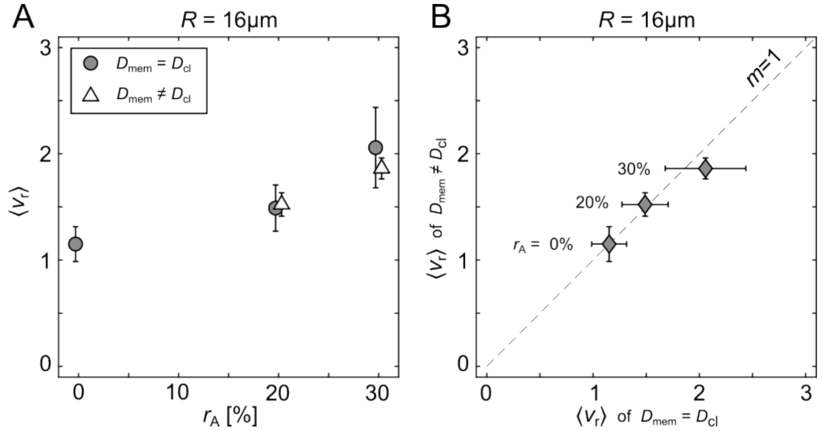

Fig. S4 Comparison of domain dynamics between diffusion properties for $R = 16$ µm. **A** shows the relation between r_A and $\langle v_r \rangle$ for the simulations with $D_{mem} = D_{cl}$ (gray circles) and $D_{mem} \neq D_{cl}$ (white triangles). **B** shows the $\langle v_r \rangle$ of $D_{mem} = D_{cl}$ and $D_{mem} \neq D_{cl}$ plotted against each other for different r_A. The dashed line shows the slope of unity.
